# Cyan fluorescent protein expression in ganglion and amacrine cells in a *thy1*-CFP transgenic mouse retina

**Published:** 2008-08-25

**Authors:** Iona D. Raymond, Alejandro Vila, Uyen-Chi N. Huynh, Nicholas C. Brecha

**Affiliations:** 1 Departments of Neurobiology and Medicine, David Geffen School of Medicine at University of California Los Angeles, Los Angeles, CA; 2 Department of Neurobiology and Anatomy, University of Texas Medical School at Houston, Houston, TX; 3 Jules Stein Eye Institute, David Geffen School of Medicine at University of California Los Angeles, Los Angeles, CA; 4 CURE Digestive Diseases Research Center, David Geffen School of Medicine at University of California Los Angeles, Los Angeles, CA; 5 Veterans Administration Medical Center-West Los Angeles, Los Angeles, CA

## Abstract

**Purpose:**

To characterize cyan fluorescent protein (CFP) expression in the retina of the thy1-CFP (B6.Cg-Tg(Thy1-CFP)23Jrs/J) transgenic mouse line.

**Methods:**

CFP expression was characterized using morphometric methods and immunohistochemistry with antibodies to neurofilament light (NF-L), neuronal nuclei (NeuN), POU-domain protein (Brn3a) and calretinin, which immunolabel ganglion cells, and syntaxin 1 (HPC-1), glutamate decarboxylase 67 (GAD_67_), GABA plasma membrane transporter-1 (GAT-1), and choline acetyltransferase (ChAT), which immunolabel amacrine cells.

**Results:**

CFP was extensively expressed in the inner retina, primarily in the inner plexiform layer (IPL), ganglion cell layer (GCL), nerve fiber layer, and optic nerve. CFP fluorescent cell bodies were in all retinal regions and their processes ramified in all laminae of the IPL. Some small, weakly CFP fluorescent somata were in the inner nuclear layer (INL). CFP-containing somata in the GCL ranged from 6 to 20 μm in diameter, and they had a density of 2636±347 cells/mm^2^ at 1.5 mm from the optic nerve head. Immunohistochemical studies demonstrated colocalization of CFP with the ganglion cell markers NF-L, NeuN, Brn3a, and calretinin. Immunohistochemistry with antibodies to HPC-1, GAD_67_, GAT-1, and ChAT indicated that the small, weakly fluorescent CFP cells in the INL and GCL were cholinergic amacrine cells.

**Conclusions:**

The total number and density of CFP-fluorescent cells in the GCL were within the range of previous estimates of the total number of ganglion cells in the C57BL/6J line. Together these findings suggest that most ganglion cells in the *thy1*-CFP mouse line 23 express CFP. In conclusion, the *thy1*-CFP mouse line is highly useful for studies requiring the identification of ganglion cells.

## Introduction

The use of transgenic mouse technology to drive the expression of reporter molecules in neurons is rapidly becoming an important tool for visualizing neuronal populations and their neural circuitry. For example, in the retina, transgenic approaches have been successfully employed for labeling specific neuronal populations by expressing reporter molecules such as β-galactosidase (β-gal), human placental alkaline phosphatase (PALP), or green fluorescent protein (GFP) and related fluorescent markers, such as cyan fluorescent protein (CFP), under the control of specific promoters that drive their expression in photoreceptors, horizontal, bipolar, amacrine, and ganglion cell types [1-6]. However, expression of the reporter molecule in retinal neurons must be carefully characterized and, in some cases, integrated with data from previous studies using cellular labeling techniques, such as Golgi and reduced silver staining, immunohistochemistry, and intracellular labeling [2,7-11], before the experimental utilization of these transgenic animals.

Classically, neuronal subclasses in the retina have been identified and categorized based on their position within the retinal matrix, morphology, physiology, and neurochemistry. The retina consists of five basic neuronal cell types: photoreceptor, horizontal, bipolar, amacrine, and ganglion cells. These cells occupy three distinct cell layers; the outer nuclear layer (ONL), inner nuclear layer (INL), and ganglion cell layer (GCL). Together, these neurons form local circuits that are segregated in two synaptic layers: the outer plexiform layer (OPL) and inner plexiform layer (IPL) [12,13]. Amacrine and displaced amacrine cells, the most diverse group of interneurons in the retina, comprise 20–30 morphologically and neurochemically distinct subclasses [14]. A few, such as the cholinergic starburst amacrine cells, have been functionally characterized [15]. About 10–12 distinct ganglion cell types occupy the GCL of the mammalian retina [16-21], and they are characterized primarily based on morphological and physiologic criteria [22,23].

In the mammalian retina, about half of the cells in the GCL are ganglion cells, while the other half are displaced amacrine cells [24-27]. Previous studies that required the differentiation of ganglion cells from other cells in the GCL have used immunohistochemistry with neurochemical markers [28-30] [31-35] or retrograde cell labeling [36,37]. A transgenic mouse line expressing an endogenous fluorescent reporter in ganglion cells would eliminate the need for surgery, which is required for retrograde ganglion cell labeling, as well as remove uncertainties inherent with retrograde transport or antibody labeling. This method would provide a reliable approach for in vivo and in vitro visualization of ganglion cells that can be positively identified for imaging and electrophysiological studies in live retinal cell cultures, slices, and explants.

This study describes in detail the pattern of CFP retinal expression in a *thy1*-CFP (#23) transgenic mouse line developed by Feng and colleagues [5], in which a genetically modified mouse *thy1* promoter drives the expression of the CFP gene in neurons [5,38]. The *Thy1* gene codes for an immunoglobulin superfamily protein that is expressed by neurons, including ganglion cells, and some non-neuronal cell types [39,40]. In the *thy1*-CFP mouse line, retinal CFP expression remains stable among individuals of the colony. It is mainly localized to ganglion cells and a subset of amacrine cells, the cholinergic amacrine and displaced amacrine cells, which can be easily distinguished based on their small size and weak CFP expression. Immunohistochemical studies with antibodies directed to neurofilament light (NF-L), neuronal nuclei (NeuN), calretinin (CR), and Brn3a, which immunolabel ganglion cells, confirm that the majority of CFP-containing cells are, in fact, ganglion cells. These findings underscore the value of the *thy1*-CFP transgenic mouse line for studies requiring the efficient identification of ganglion cells for in vivo and in vitro studies.

## Methods

### Animal procedures

Adult *Thy1*-CFP C57BL/6J transgenic mice, from line B6.Cg-Tg(Thy1-CFP)23Jrs/J [5], were purchased from the Jackson Laboratory (Bar Harbor, ME). Mice were housed and bred in the Animal Care Facility at the David Geffen School of Medicine at the University of California, Los Angeles (Los Angeles, CA), 12 h light-dark cycle, with chow and water ad libitum. All animal guidelines of the National Institutes of Health, the Association for Research in Vision and Ophthalmology, and the University of California, Los Angeles were followed concerning animal welfare. Mice used for tissue collection for immunohistochemistry and PCR were euthanized by isofluorane (Novaplus, Lake Forest, IL) inhalation anesthesia and decapitated. 500 μl to 1 ml of 100% isofluorane was allowed to vaporize in an enclosed chamber and several animals were sacrificed.

### Thy1-CFP PCR

*Thy1*-CFP mice were genotyped to confirm the inclusion of the CFP reporter transgene under the control of the *thy1* promoter. Mouse tail DNA was prepared by digesting 2–3 mm of mouse tail overnight at 55 °C with 20 μl of proteinase K (10 mg/ml) in 180 μl tail digestion buffer: 50 mM Tris-HCl, pH 8.0, 1 mM MgCl_2_, 1% Tween-20. Genotypes were determined by PCR using the following primers: Thy1F1 (TCT GAG TGG CAA AGG ACC TTA GG) from *thy1* sequence and ECFPR1 (CCG TCG CCG ATG GGG GTG TT) for *thy1-CFP* mice. PCR reactions were performed in 25 μl total volume containing 50 mM Tris-HCl (pH 9.2), 16 mM ammonium sulfate, 3.5 mM MgCl_2_, 0.1% Tween-20, 0.2 mM dNTP, 2.5 U KlentaqLA (Clonetech, Mountain View, CA), and 1 μl mouse tail DNA. The PCR reaction was started at 94 °C for 1.5 min, and then continued for 35 cycles as follows: 94 °C for 30 s,, 60 °C for 60 s, and 72 °C for 60 s, with a final amplification step of 72 °C for 10 min. Approximately one-third of the PCR reaction was separated by electrophoresis in 1.5% agarose, stained with 1.25 μg/ml ethidium bromide in 1X Tris-Acetate-EDTA buffer (TAE) and photographed. The animals were scored as CFP positive if the predicted 173 bp PCR DNA product was obtained.

### Tissue preparation

After mice were euthanized, their eyes were removed and dissected. For transverse sections, the eye cups containing retinas were fixed in 4% paraformaldehyde in 0.1 M phosphate buffer (PB), pH 7.4, for 15 to 60 min at room temperature and then transferred to 30% sucrose overnight at 4 °C. The eyecups with the retina were frozen in ornithine carbamyl transferase (OCT; Reichert-Jung, Bensheim, Germany), sectioned perpendicularly to the vitreal surface at 10–12 μm with a cryostat, and retinal sections were collected onto gelatin-coated slides and stored at −20 °C until antibody staining. For wholemount preparations, retinas were removed from the eyecup after 1 min in 4% paraformaldehyde, dissected, and flattened between two slides with spacers on both ends and fixed for an additional 15 to 30 min. Isolated retinas were transferred to 0.1 M PB and stored at 4 °C until antibody staining.

### Immunohistochemistry

Retinal sections were rinsed in 0.1 M PB for 30 min, then incubated in a humidified chamber for 12–16 h at 4 °C in the primary antibody solution. Primary antibody solution routinely contained 1%–5% normal goat serum, 1% bovine serum albumin, and 0.5% Triton X-100 in 0.1 M PB, pH 7.4. Retinal sections were washed and incubated in secondary antibodies conjugated with 1:1000 Alexa 568 or Alexa 633 (Molecular Probes, Eugene, OR) for 1–2 h at room temperature in 0.1 M PB containing 0.5% Triton X-100. Sections were washed for 30 min with 0.1 M PB, air-dried, and mounted using the ProLong Antifade Kit (Molecular Probes). Retinal wholemounts were incubated in 1% sodium borohydride (in deionized water) for 1 h at room temperature, rinsed and incubated in primary antibody solution as described for retinal sections, for 5–7 days at 4 °C. Retinal wholemounts were washed and incubated in secondary antibody solution, as described for retinal sections, for 1–2 days at 4 °C. Wholemounts were rinsed again, mounted ganglion cell side up onto glass slides, air dried, then coverslipped using the ProLong Antifade Kit or Vectashield Mounting Medium (Vector Laboratories, Burlingame, CA) containing the fluorescent nuclear dye 4’,6-diamidino-2-phenylindole (DAPI).

### Antibodies

Primary antibodies that immunolabel amacrine and displaced amacrine cells and ganglion cells were included: mouse monoclonal antibodies against syntaxin-1 (HPC-1; Sigma, St Louis, MO), L-glutamate decarboxylase_67_ (GAD_67_; Millipore, Temecula, CA), neuronal nuclei (NeuN; Millipore), the POU-domain protein, Brn3a (clone 5A3.2; Millipore), calretinin (CR; Millipore), and glycine transporter −1 (GlyT-1; Millipore), rabbit polyclonal antibodies against the GABA plasma membrane transporter-1 (GAT-1; Millipore), and neurofilament light (NF-L; Millipore), and goat polyclonal antibodies to choline acetyltransferase (ChAT; Millipore). Immunolabeling with these primary antibodies was visualized using fluorochrome-conjugated secondary antibodies Alexa 568 and Alexa 633 conjugated goat-antirabbit IgG, donkey antigoat IgG, goat antimouse IgG (Molecular Probes). Controls, including cognate peptide adsorption studies, were performed to evaluate specificity of the primary antibodies and binding of the secondary antisera to the appropriate antigen-antibody complex. These preparations were evaluated using both conventional and confocal microscopy.

### Light and confocal microscopy

Retinal sections and wholemounts were examined and analyzed with a Zeiss LSM 510 Meta confocal microscope (Zeiss, Oberkochen, Germany) equipped with argon, helium, and neon lasers, using a Plan-Neofluar 40x 1.3 n.a. or a Plan-Neofluar 63x1.25 n.a. objective, or an Axiocam digital camera (Carl Zeiss, Inc., Thornwood, NY) mounted onto a Zeiss Axioplan Two fluorescence microscope (Carl Zeiss, Inc.). Digital images were acquired at a magnification zoom of 1X to 2.5X, and a resolution of 1024×1024 or 2048×2048. Most confocal images were acquired at an optical thickness between 0.5 μm and 1.0 μm and ~1.0 Airy Unit. For projections, typically 3–8 optical sections were acquired with an average total thickness of 3–8 μm and compressed for viewing. Digital confocal images were saved as Zeiss .LSM files, and final publication quality images were exported in the .TIFF format at 300 dpi using Zeiss LSM 510 Meta software version 3.2 (Zeiss Ltd., Thornwood, NY). Images were adjusted for contrast and brightness, labeled, and formatted using Adobe Photoshop 7.0.1 (Adobe Systems, Inc., San Jose, CA) and saved at 300 dpi at their final magnification.

### Quantitative analysis

Average somal diameters and densities of CFP-expressing cells were determined from stacked images of confocal optical sections of the GCL or the INL taken from retinal wholemounts. Images were acquired every 500 μm from the optic nerve head along the dorsoventral and nasotemporal axes, and 200×200 μm fields were analyzed in their entirety. Somal size was obtained by averaging the maximum and minimum diameters of CFP fluorescent cells measured using Zeiss LSM 510 software (Zeiss Ltd.). We did not attempt to correct for the negligible shrinkage of the tissue from the mounting process. The number of NF-L, NeuN, CR, Brn3a, HPC-1, GAT-1, GAD_67_, and ChAT immunoreactive cell bodies containing CFP fluorescence were determined from similar digital images taken 1.5 mm from the optic nerve head in the nasal retina along the nasotemporal axis.

## Results

In the *thy1*-CFP (#23) mouse line, CFP expression is driven by the mouse thy1.2 promoter sequence, genetically modified for neuronal expression [5]. This line was developed by the Sanes and Lichtman groups [5], and it was provided to the Jackson Laboratory for distribution. CFP expression is in neurons distributed to different regions of the neuroaxis, including cortex, cerebellum, and spinal cord, in a mouse perfused transcardically with 4% paraformaldehyde and the brain processed by standard techniques [36]. Feng et al. reported CFP expression along the neural axis, excluding the retina [5].

### Cyan fluorescent protein expression in the *thy1*-CFP mouse retina

**Figure 1:**
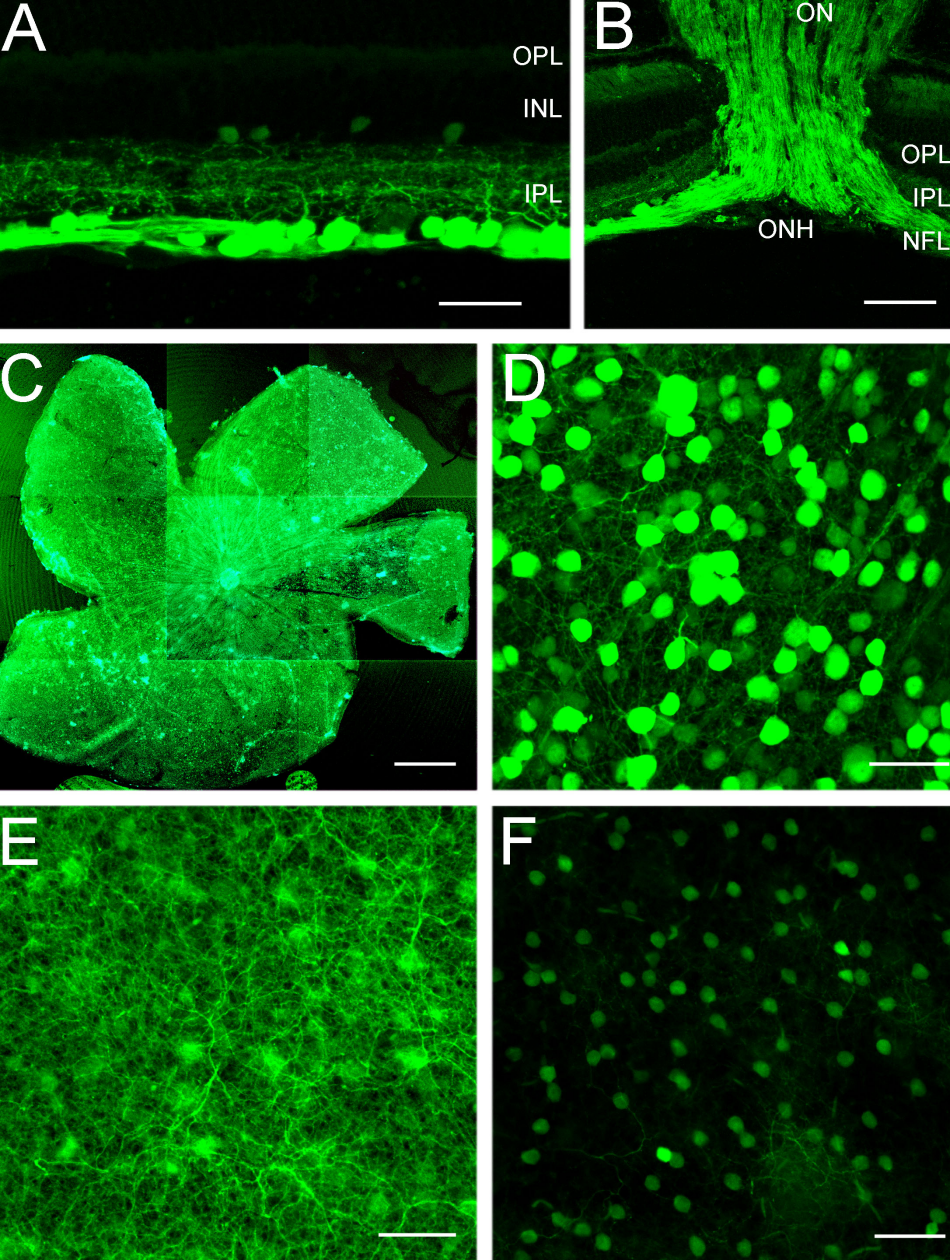
Cyan fluorescent protein expression in the retinas of *thy1*-CFP transgenic mice in confocal images of transverse sections **(A,B)** and wholemounts **(C**-**F)**. **A:** Most cyan fluorescent protein (CFP) expression is localized to brightly fluorescent cell bodies in the ganglion cell layer (GCL), processes that form a dense plexus in all laminae of the inner plexiform layer (IPL) and axons in the nerve fiber layer (NFL). Small, weakly CFP fluorescent cells are in the proximal inner nuclear layer (INL) and the GCL. The scale bar represents 35 µm. **B:** CFP expression is prominent in ganglion cell axons in the NFL, optic nerve head (ONH), and optic nerve (ON). Scale bar equals 60 µm. **C:** Low-magnification composite image of a wholemount shows CFP expression in all retinal regions. Scale bar equals 550 μm. **D:** CFP expression is localized to brightly and weakly fluorescent cell bodies of various sizes in the GCL. Image is taken in midperipheral nasal retina, 1.5 mm from the optic nerve head. **E:** A plexus of CFP-containing processes occupies the IPL. Figure E shows the same region as in **D**. **F:** Weak CFP expression is evident in small cell bodies in the proximal INL. Panel **F** shows the same region as in **D**. Scale bar for **D**-**F** equals 35 μm. In **A** and **B**, outer plexiform layer is abbreviated OPL.

CFP expression was limited exclusively to the inner retina, and it was primarily localized to numerous cell bodies in the GCL and their dendrites in the IPL. CFP fluorescence in these cells ranged in intensity from weak to very strong. In addition, there were weakly fluorescent small somata in the INL and GCL (Figure 1A). The processes of CFP fluorescent cells ramified extensively in all laminae of the IPL (Figure 1A), and fluorescent axonal labeling was evident in the nerve fiber layer, optic nerve head, and optic nerve (Figure 1B). CFP expression was not observed in the outer retina, including the outer plexiform and nuclear layers (OPL; ONL).

CFP-containing cell bodies were distributed across the entire retina (Figure 1C), and no regional differences in their distribution were observed in either wholemount preparations or vertical sections that included peripheral and central retina. The majority of CFP-expressing cells occupied the GCL (Figure 1D). These cells varied in size and in the intensity of CFP fluorescence. In addition, small somata weakly expressing CFP were in the proximal INL, near the IPL (Figure 1F) in all retinal regions. CFP-positive processes ramified densely throughout all laminae of the IPL (Figure 1E), with the densest plexus of fibers in laminae 2 and 4 of the IPL. CFP-containing processes had numerous varicosities in all laminae of the IPL. Due to the high density of fluorescent cells, especially in the GCL, however, it was difficult to determine with certainty which processes originated from individual somas.

**Figure 2:**
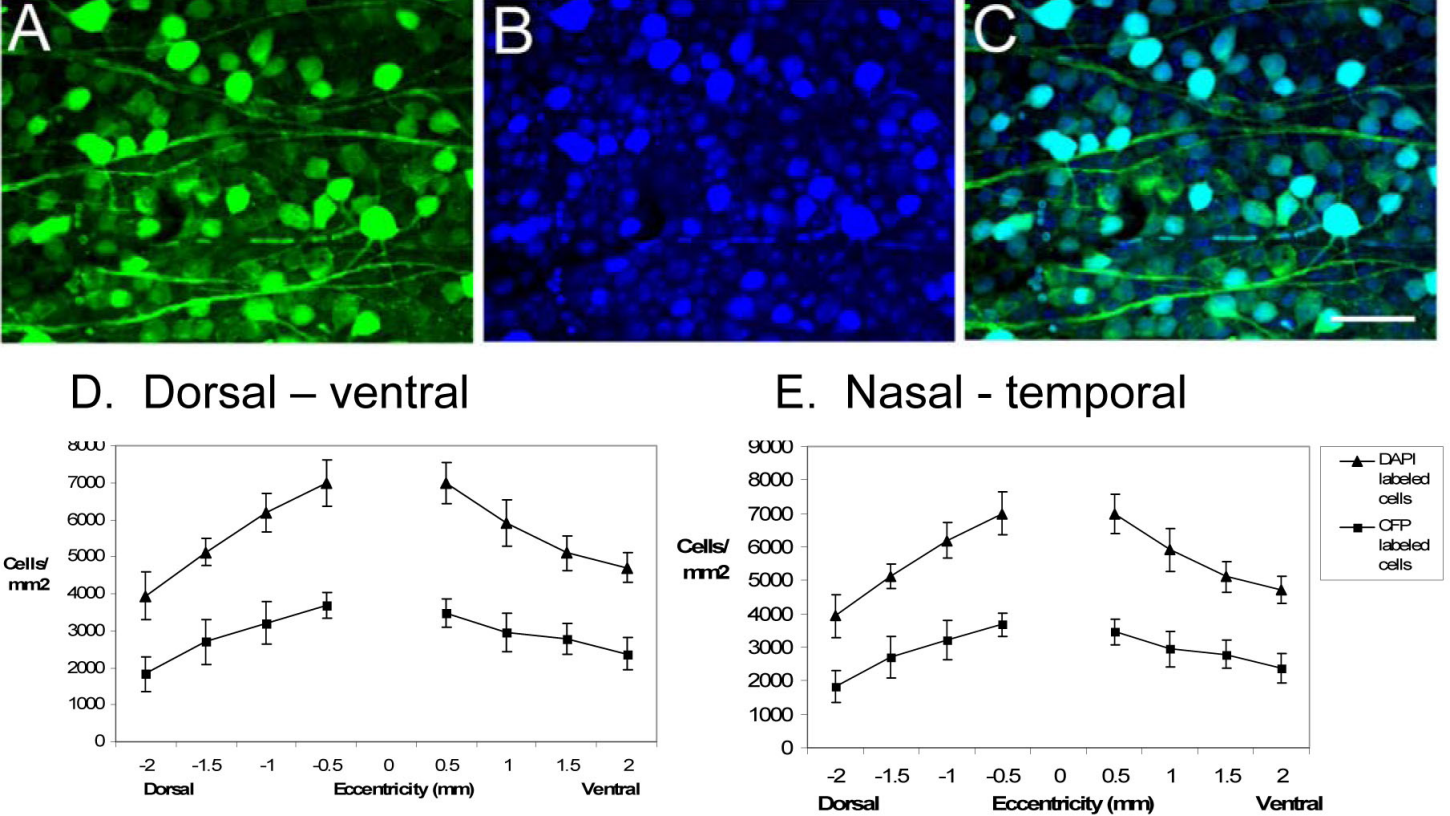
Comparison of cyan fluorescent protein expression in the ganglion cell layer with DAPI-labeled retinal neurons. **A:** Image of a retinal wholemount through the midperipheral ganglion cell layer (GCL) showing that cyan fluorescent protein (CFP) expression is localized to numerous cell bodies. **B:** Image illustrates the same region as in **A**, showing DAPI fluorescence in all cell nuclei in the GCL. **C:** A merged image of **A** and **B** shows colocalization of CFP expression and DAPI nuclear labeling in about half of the neuronal nuclei in the GCL. Scale bar for **A**-**C** represents 50 μm. Graphs showing the average densities of CFP- and DAPI-labeled cells in the GCL along the dorsoventral (**D**) and nasotemporal (**E**) axes of five retinal wholemounts from five different thy1-CFP transgenic mice. Error bars are SEM.

The density of CFP-expressing cells in the GCL was determined, from five retinal wholemounts, along the nasotemporal and dorsoventral axes bisecting the optic nerve head (Figure 2D-E). Areas of 200 200 μm^2^ were sampled at 500 μm intervals from the optic nerve head, and all CFP-labeled cells were included regardless of fluorescence intensity and size. The cell density in the GCL ranged from 1827±280 to 3589±470 cells/mm^2^ along the dorsoventral axis from central to peripheral retina. Along the nasotemporal axis from central to peripheral retina, the cell density ranged from 2478±356 to 3243±281 cells/mm^2^. To determine what percentage of total cells in the GCL is CFP-positive, we compared the number of cells labeled with the fluorescent nuclear dye, DAPI, with the number of CFP-expressing cells in confocal images (Figure 2A-C). DAPI-labeled glia and endothelial cells were excluded from the counts based on the distinct morphology of their nuclei. Counts of the number of DAPI- and CFP-expressing somata in the GCL, 1.5 mm nasal from the optic nerve head, showed that virtually all (99.7%) of the CFP-expressing cells contained DAPI, as expected. Conversely, about half (51.2%) of the DAPI labeled cells expressed CFP in the GCL. On average, 51.9±3.1% of all neurons in the GCL contained CFP at all retinal eccentricities.

**Figure 3:**
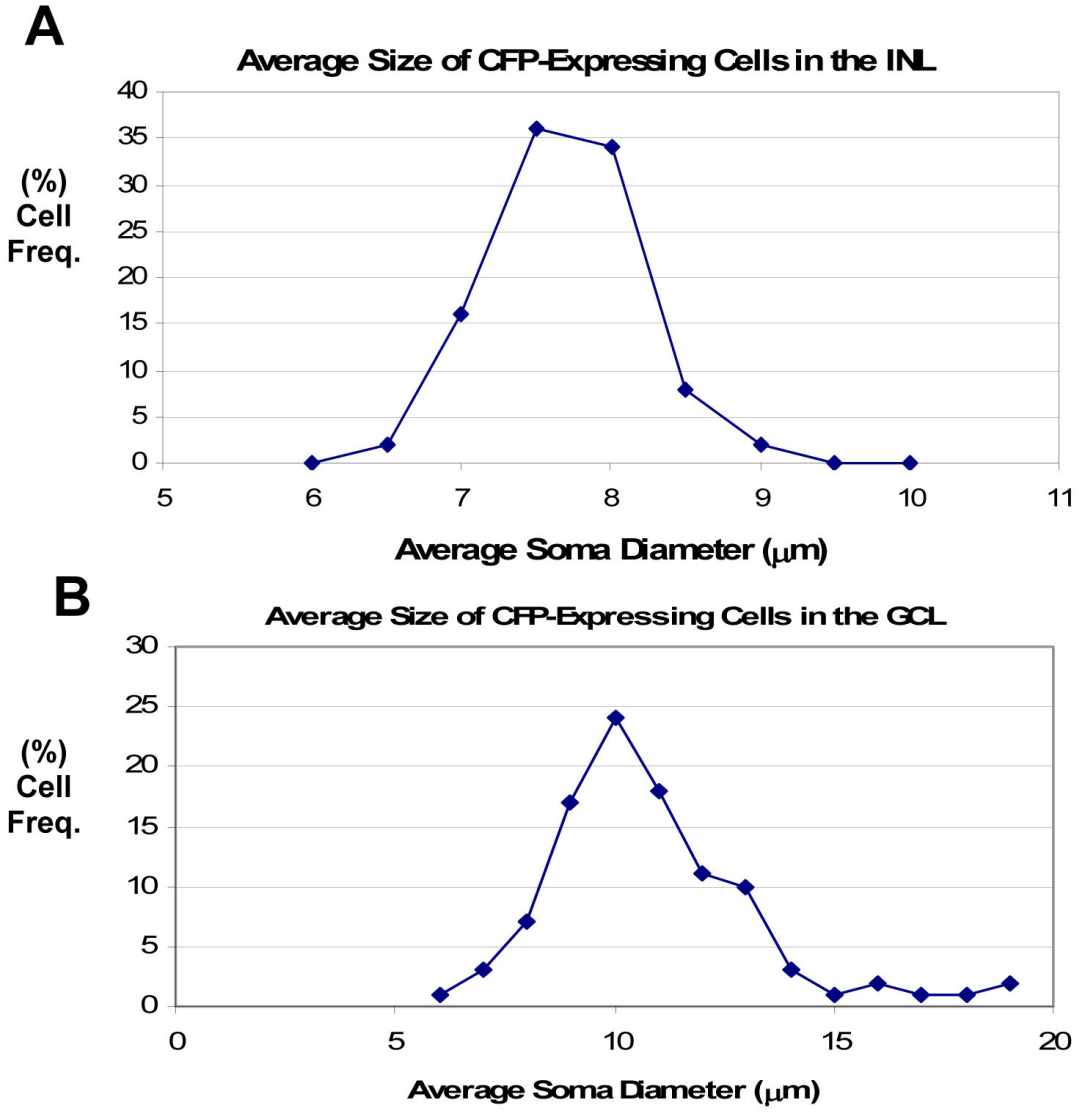
Graphs showing the percent cell frequency of average somal diameters of cyan fluorescent protein-expressing cells in the ganglion cell layer (**A**) and inner nuclear layer (**B**). **A:** The somal diameter in the ganglion cell layer (GCL) averaged 11.30±2.05 µm (n=250). **B:** The somal diameter in the inner nuclear layer (INL) averaged 7.91±0.48 µm (n=50).

The average somal diameter of CFP-expressing cells was measured in the GCL and INL in the midperipheral nasal retina at 1.5 mm from the optic nerve head (Figure 3). In the GCL, somal diameter averaged 11.30±2.05 µm (n=250), and somal diameters ranged from 6.12 to 19.74 µm. All CFP-expressing cells were included in this measurement, regardless of the level of CFP fluorescence intensity. In the INL, somal diameter averaged 7.91±0.48 µm (n=50), and somal diameters ranged from 6.32 to 10.25 µm. All CFP-expressing cells in the INL were characterized by a small somal diameter and weak CFP fluorescence. A comparison of cell density and somal diameters obtained from each of the five retinal wholemounts in the GCL and INL indicated that there was no significant variability in the number, density, or size of CFP immunoreactive cells from animal to animal (data not shown).

### Cyan fluorescent protein expression in ganglion cells

Extensive CFP expression in the GCL, nerve fiber layer, optic nerve head, and optic nerve indicated that the majority of fluorescent marker protein in the *thy1*-CFP mouse retina was localized in ganglion cells. Therefore, previously characterized and commonly used neurochemical markers for ganglion cells, including NF-L, NeuN, Brn3a, and CR, were used to confirm the CFP-containing cells in the GCL in ganglion cells.

**Figure 4:**
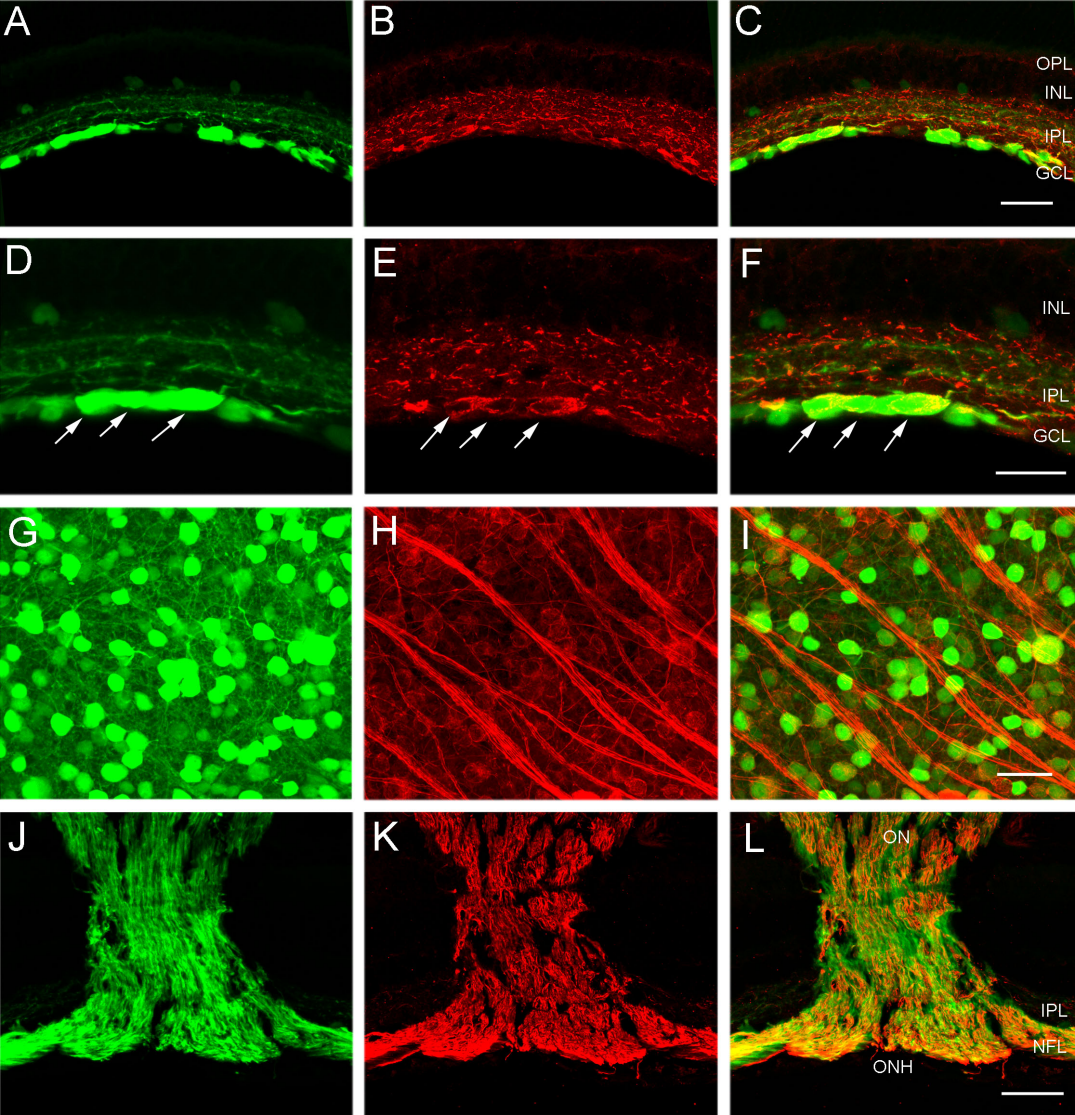
CFP-containing ganglion cells express NF-L immunoreactivity in their soma, dendrites and axons in the fiber layer, optic nerve head and optic nerve. **A:** Transverse section of peripheral retina shows cyan fluorescent protein (CFP) expression in numerous cell bodies in the ganglion cell layer (GCL). **B:** Same section as in **A** shows neurofilament light (NF-L) immunoreactivity in ganglion cell somata in the GCL and dendrites in the inner plexiform layer (IPL). **C:** A merged image of **A** and **B** demonstrates colocalization of CFP expression and NF-L immunoreactivity in many cell bodies in the GCL and dendrites in the IPL. The scale bar for **A**-**C** is 40 μm. **D:** A higher magnification image of transverse retina shows CFP fluorescence in ganglion cells, including large ganglion cell somata (arrows). **E:** NF-L immunoreactivity is evident in numerous cell bodies in the GCL including the large ganglion cell somata (arrows). **F:** A merged image of **D** and **E** demonstrates colocalization of CFP expression and NF-L immunoreactivity in most cell bodies in the GCL and dendrites in the IPL. The scale bar for **D**-**F** is 25 μm. **G:** CFP is localized to brightly and weakly fluorescent cell bodies of various sizes in the GCL. Image is from a retinal wholemount located 1.5 mm from the optic nerve head in midperipheral nasal retina. **H:** NF-L immunoreactivity is evident in ganglion cell somata in the same region as **G**. **I:** A merged image of **G** and **H** shows colocalization of CFP expression and NF-L immunoreactivity in ganglion cell bodies. The scale bar for **G**-**I** is 30 μm. **J:** CFP fluorescence is evident in ganglion cell axons in the fiber layer, optic nerve head and optic nerve, in this low magnification image of a transverse section through the optic nerve head. **K:** The same section as in **J**, shows NF-L immunoreactivity in ganglion cell axons. **L:** A merged image of **J** and **K** shows colocalization of CFP and NF-L immunoreactivity in most ganglion cell axons in the fiber layer, optic nerve head and optic nerve. Note: there is not complete overlap of CFP and NF-L in ganglion cell axons, as some NF-L labeled cells do not express CFP. The scale bar for **J–L** is 140 μm. In **C** and **F** inner nuclear layer is abbreviated INL, and outer plexiform layer is abbreviated OPL.

NF-L is an intermediate filament protein that is prominently expressed in mature neurons. In the mammalian retina NF-L antibodies immunolabel close to 90% of all ganglion cells [28-30]. In transverse sections of the *thy1*-CFP mouse retina, NF-L immunoreactivity was characterized by prominent immunostaining that was restricted primarily to the inner retina; weak NF-L immunoreactivity was observed in ganglion cell bodies, and immunostaining was characterized by a reticular appearance. In addition, NF-L immunoreactivity was localized to dendrites in all laminae of the IPL and to individual axons that formed thick bundles in the fiber layer that became increasingly frequent in central retina (Figure 4B,E). This pattern of NF-L expression was similar to that described in earlier studies [28-30].

CFP expression colocalized with NF-L immunoreactivity in ganglion cell bodies in the GCL (Figure 4C,F) and dendrites in the IPL. In addition, there were some CFP-containing processes that did not contain NF-L immunoreactivity. In wholemounts, NF-L immunoreactivity in the GCL was found in numerous somata that varied in size from small to very large, and in the optic fiber layer immunolabeled fiber bundles (Figure 4H) converged in a radial pattern at the optic nerve head (Figure 4L). Colocalization of CFP and NF-L immunoreactivities was determined from confocal images of the GCL from five retinal wholemounts (Table 1). About 60% of the CFP-expressing cells were NF-L immunoreactive, while 70% of NF-L immunoreactive cell bodies in the GCL also contained CFP. Finally, colocalization of CFP expression and NF-L immunoreactivity was evident in ganglion cell axons in the nerve fiber layer, optic nerve head, and optic nerve (Figure 4J-L).

**Table 1:** Summary of colocalization of cyan fluorescent protein-expressing cells in the GCL with ganglion and amacrine cell markers.

**Marker**	**Labeled cells**	**CFP positive cells**	**Total colocalized cells**	**Percent CFP cells immunolabeled**	**Percent immunolabeled cells with CFP**
DAPI (GCL)	1243	638	636	0.997	0.512
NF-L (GCL)	710	821	506	0.616	0.713
NeuN (GCL)	1291	782	746	0.954	0.578
CR (GCL)	1013	697	630	0.904	0.622
CR (INL)	1193	312	284	0.91	0.238
Brn3a (GCL)	537	832	512	0.615	0.953
HPC-1 (GCL)	612	563	54	0.096	0.088
GAD67 (GCL)	587	1239	154	0.124	0.262
GAT-1 (GCL)	509	946	87	0.092	0.171
ChAT (GCL)	255	1251	255	0.204	1
ChAT (INL)	318	358	311	0.867	0.978

The colocalization of cyan florescent protein (CFP)-expressing cells with ganglion and amacrine cell markers was determined in the ganglion cell layer (GCL), and the inner nuclear layer (INL). Neurofilament light (NF-L), neuronal nuclei (NeuN), calretinin (CR), and a POU-domain protein (Brn3a) were used to immunolabel ganglion cells. Syntaxin 1a (HPC-1), glutamate decarboxylase 67 (GAD_67_), GABA plasma membrane transporter-1 (GAT-1), choline acetyltransferase (ChAT), and in some cases CR, were used to label amacrine and displaced amacrine cells. DAPI (4',6-diamidino-2-phenylindole) was used to label all retinal nuclei.

**Figure 5:**
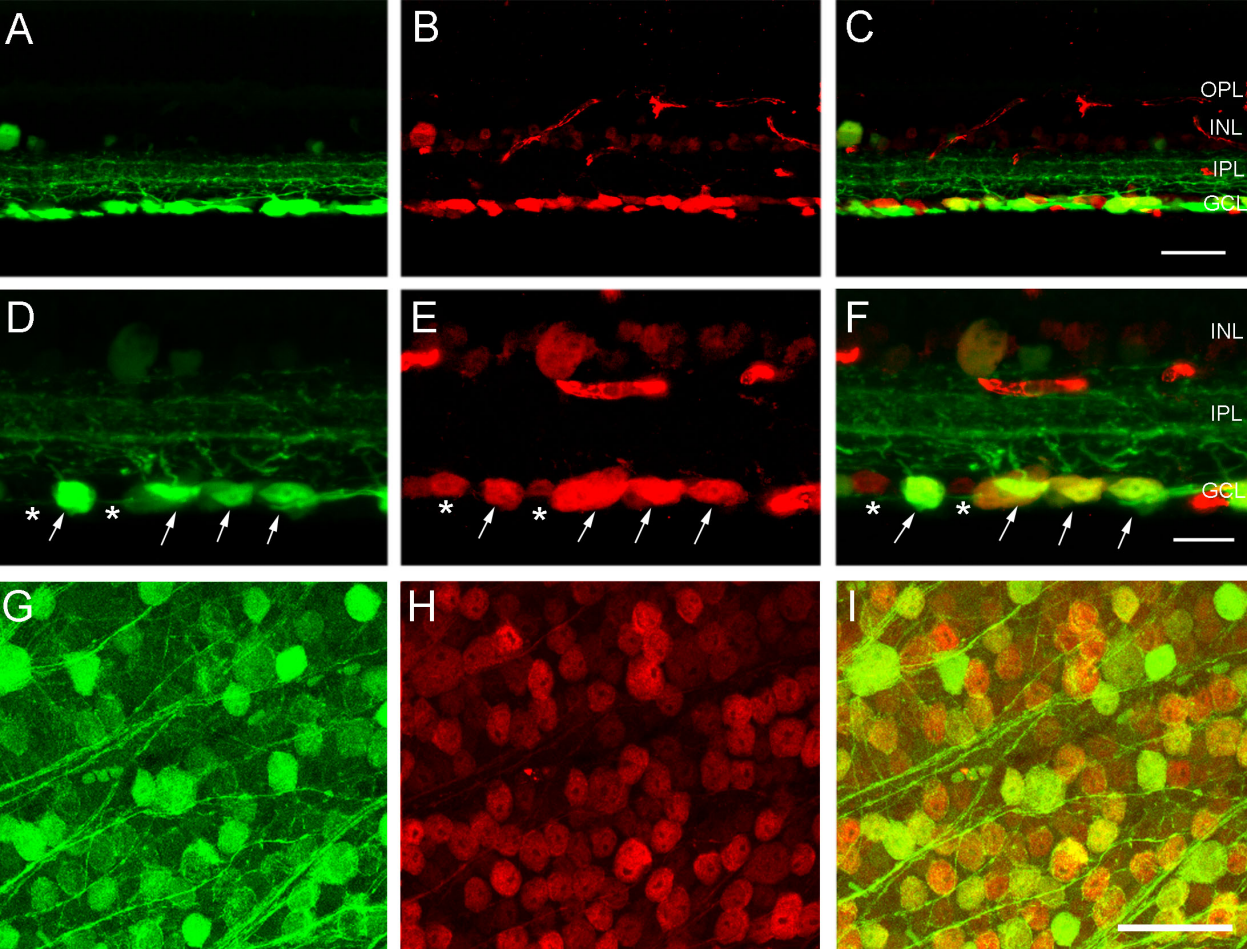
CFP-containing ganglion cells express NeuN immunoreactivity, a marker commonly used to identify retinal ganglion cells. **A:** Transverse section of peripheral retina shows cyan fluorescent protein (CFP) expression in numerous cell bodies in the ganglion cell layer (GCL). **B:** Neuronal nuclei (NeuN) immunoreactivity is localized in ganglion cell somata in the GCL (and weak immunoreactivity is in cell bodies in the inner nuclear layer; INL). **C:** A merged image of **A** and **B** shows colocalization of CFP expression and NeuN immunoreactivity in most cell bodies in the GCL. The scale bar for **A–C** is 45 μm. **D:** A higher magnification image shows CFP fluorescence in large ganglion cells (arrows) in the GCL. **E:** The same section as in **D** shows NeuN immunoreactivity in numerous ganglion cell somata in the GCL (arrows). The small, weakly NeuN-immunoreactive cells are displaced amacrine cells (stars). **F:** A merged image of **D** and **E** shows colocalization of CFP and NeuN immunoreactivity in ganglion cells (arrows) and a lack of colocalization of CFP and NeuN immunoreactivity in displaced amacrine cells (stars). The scale bar for **D**-**F** is 25 μm. **G:** CFP is localized to brightly and weakly fluorescent cell bodies of various sizes in the GCL. The image is from a retinal wholemount located 1.5 mm from the optic nerve head in midperipheral nasal retina. **H:** The same region as in **G** shows NeuN immunoreactivity in numerous cell somata in the GCL, including ganglion cells and displaced amacrine cells. **I:** The merged image of **G** and **H** shows colocalization of CFP and NeuN immunoreactivity in numerous ganglion cell somata in the GCL. The scale bar for **G**-**I** is 45 μm. In **C** and **F** inner plexiform layer is abbreviated IPL, and outer plexiform layer is abbreviated outer plexiform layer (OPL).

Numerous investigations, including neurodegenerative studies, have identified retinal ganglion cells using a monoclonal antibody to NeuN, a neuronal specific nuclear protein of unknown function [31-35]. NeuN labeling has been suggested as a particularly useful reagent for quantifying the loss of retinal ganglion cells, since NeuN immunostaining is mainly restricted to the cell soma and is easily identifiable [35]. NeuN immunoreactivity was localized to numerous cells in the proximal INL and GCL of varied size and intensity of immunostaining (Figure 5B,E). NeuN immunoreactivity was absent from photoreceptors and bipolar cells, and from any processes in the IPL and OPL or the nerve fiber layer. The expression of NeuN is similar to that previously reported in the mammalian retina [31-35].

CFP expression colocalized with NeuN immunoreactivity in numerous somata in the GCL (Figure 5). In addition, there were NeuN immunoreactive cells that did not contain CFP (Figure 5D-F). Colocalization of CFP and NeuN immunoreactivities was determined from confocal images of the GCL from five retinal wholemounts (Table 1). About 95% of the CFP-expressing cells were NeuN immunoreactive, while 57.8% of the NeuN immunoreactive cell bodies in the GCL contained CFP.

The colocalization of CFP expression with other well established retinal ganglion cell markers, including CR and Brn3a [41-45], was also evaluated (Table 1). CR, a 29 kD calcium-binding protein closely related to calbindin, was extensively expressed in numerous somata in the GCL and INL, as previously described [41-43]. CR immunoreactive somata varied in size, with mainly small somata in the INL and small, medium, and large somata in the GCL. In the GCL, CFP expression colocalized with CR immunoreactivity in 90.4% of the CFP cells, while 62.2% of the CR immunostained cells contained CFP. In the INL, 91.0% of the CFP cells contained CR immunoreactivity and 23.8% of the CR immunolabeled cells expressed CFP.

Antibodies to Brn3a, a POU domain transcription factor, have also been extensively used in studies to label ganglion cells [44,46]. Brn3a immunoreactive nuclei were numerous in the GCL (data not shown). CFP colocalized with Brn3a immunoreactivity in 61.5% of the CFP cells in the GCL, while 95.3% of the Brn3a immunoreactive cells contained CFP (Table 1).

### Cyan fluorescent protein expression in amacrine cells

CFP expression in small somata in the proximal INL and GCL indicated that some retinal CFP expression in the *thy1*-CFP transgenic mouse line was localized to amacrine and displaced amacrine cells. Therefore, we used well established neurochemical markers to test if the small CFP-containing somata were amacrine and displaced amacrine cells.

**Figure 6:**
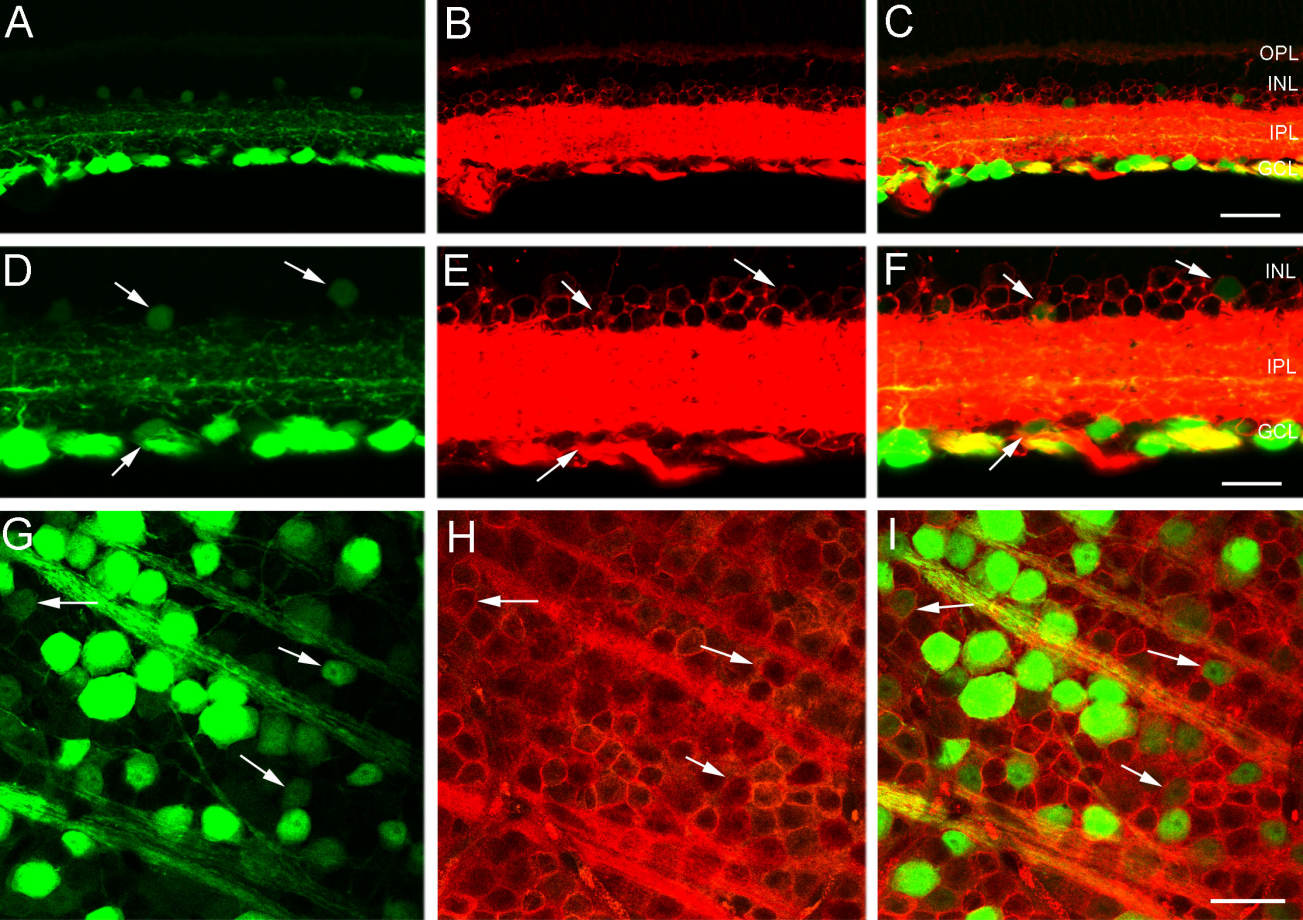
Small, weak CFP fluorescent somata in the INL and GCL contain HPC-1 immunoreactivity, a marker of amacrine and displaced amacrine cells. **A:** A transverse section of peripheral retina shows cyan fluorescent protein (CFP) expression in numerous somata in the ganglion cell layer (GCL) and weak CFP-expressing somata in the proximal inner nuclear layer (INL). **B:** Same section as in **A** shows syntaxin 1a (HPC-1) immunoreactivity in amacrine and displaced amacrine cell somata in the INL and GCL, respectively. HPC-1 immunostaining is very strong in the inner plexiform layer (IPL) and the weaker HPC-1 immunoreactive somata in the GCL tend to be obscured in transverse sections. **C:** A merged image of **A** and **B** shows colocalization of CFP and HPC-1 immunoreactivity in small, weakly CFP-expressing cell bodies in the INL and GCL. The scale bar for **A**-**C** is 40 μm. **D:** A higher magnification image of transverse retina shows CFP fluorescence in ganglion cells in the GCL and in smaller, weakly CFP fluorescent cells in the INL and GCL (arrows). **E:** The same section as in **D** shows HPC-1 immunoreactivity in numerous amacrine and displaced amacrine cell somata in the INL and GCL (arrows). **F:** A merged image of **D** and **E** shows colocalization of CFP expression and HPC-1 immunoreactivity in amacrine and displaced amacrine cells (arrows) in the INL, and GCL. The scale bar for **D–F** is 25 μm. **G:** CFP is localized to brightly and weakly fluorescent cell bodies of various sizes in the GCL. Image is from a retinal wholemount located 1.5 mm from the optic nerve head in midperipheral nasal retina. **H:** Same region as in **G** shows HPC-1 immunoreactivity in numerous cell somata in the GCL. **I:** A merged image of **G** and **H** shows the colocalization of CFP and HPC-1 immunoreactivity in displaced amacrine cell bodies in the GCL (arrows). The scale bar for **G**-**I** is 30 μm. In **C** and **F** outer plexiform layer is abbreviated outer plexiform layer (OPL).

Initially, the general amacrine and displaced amacrine cell marker HPC-1 (syntaxin 1a) was used to evaluate the extent of CFP expression in amacrine cells. In the *thy1*-CFP mouse retina, HPC-1 immunofluorescence labeled the inner retina robustly; it was localized primarily to a dense plexus of processes throughout all laminae of the IPL, and it faintly immunostained the cytoplasm of amacrine and displaced amacrine cell somata in the proximal INL and GCL, respectively (Figure 6B,E) [47-49]. The HPC-1 immunostaining pattern was similar to earlier reports of HPC-1 immunoreactivity in the mouse retina [47-49].

HPC-1 immunoreactivity colocalized with all of the small and weakly CFP-fluorescent cells in the proximal INL. Many of the small and weakly CFP-fluorescent cells in the GCL also contained HPC-1 immunoreactivity (Figure 6F). These cells were characterized by a round shape and, in most cases, these cells could be readily distinguished from the larger and brighter CFP-fluorescent ganglion cells. The majority of HPC-1 immunoreactive amacrine and displaced amacrine cell bodies, however, did not contain CFP, indicating that most CFP expression is mainly localized to ganglion cells. Cell counts from wholemount preparations of the *thy1*-CFP mouse retina revealed that 8.8% of the HPC-1 immunoreactive amacrine cell somata in the GCL contained CFP, while 9.6% of the total CFP-expressing cells in the GCL were HPC-1 immunoreactive displaced amacrine cells (Table 1).

CFP-expressing amacrine and displaced amacrine cells were further classified using neurochemical markers that distinguish among the different amacrine cell classes [50]. In the mammalian retina about half of the amacrine cell population is GABAergic, while the other half is glycinergic [50]. Therefore, the next set of experiments evaluated the expression of neurochemical markers for GABAergic and glycinergic amacrine and displaced amacrine cells.

**Figure 7:**
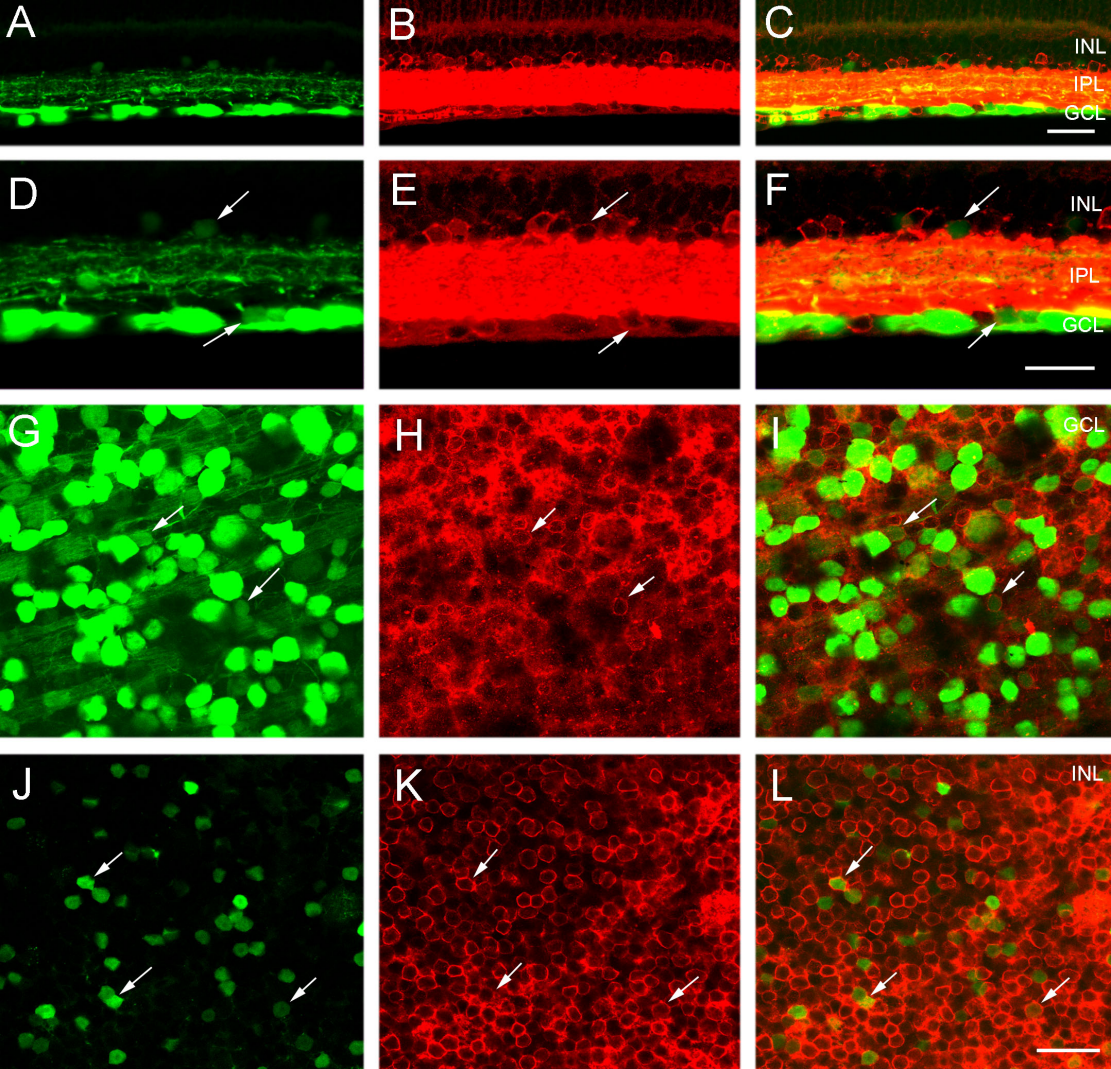
CFP expression colocalizes with GAD_67_ or GAT-1 immunoreactivity in the inner retina. **A:** A transverse section of peripheral retina shows cyan fluorescent protein (CFP) in numerous cell bodies in the ganglion cell layer (GCL), and weakly CFP-expressing cell bodies in the proximal inner nuclear layer (INL). **B:** The same region as in **A** shows L-glutamate decarboxylase 67 (GAD_67_) immunoreactivity in amacrine and displaced amacrine cell somata in the INL and GCL, respectively. **C:** The merged image of **A** and **B** shows colocalization of CFP expression and GAD_67_ immunoreactivity in small, weakly CFP-expressing cell bodies in the INL and GCL. The scale bar for **A–C** is 45 μm. **D:** A higher magnification image of transverse retina shows CFP in ganglion cell somata in the GCL and in smaller, weakly CFP fluorescent somata in the GCL and INL (arrows). **E:** GAD_67_ immunoreactivity is in numerous amacrine and displaced amacrine cell somata in the INL and GCL (arrows). **F:** The merged image of **D** and **E** shows colocalization of CFP expression and GAD_67_ immunoreactivity in amacrine and displaced amacrine cells (arrows) in the INL and GCL. The scale bar for **D**-**F** is 25 μm. **G:** CFP is localized to brightly and weakly fluorescent cell bodies of various sizes in the GCL. Image is from a retinal wholemount located 1.5 mm from the optic nerve head in midperipheral nasal retina. **H:** The same region as in **G** shows GABA plasma membrane transporter-1 (GAT-1) immunoreactivity near or at the plasma membrane of displaced amacrine cell somata in the GCL. **I:** Merged image of **G** and **H** shows colocalization of CFP expression and GAT-1 immunoreactivity in displaced amacrine cell somata in the GCL (arrows). Most GAT-1 immunoreactive somata lack CFP. **J:** CFP is localized to weakly fluorescent cell bodies in the INL. Image is from a retinal wholemount located 1.5 mm from the optic nerve head in midperipheral nasal retina. **K:** Same region as in **J** shows GAT-1 immunoreactivity in amacrine cell somata in the INL. **L:** Merged image of **J** and **K** shows colocalization of CFP expression and GAT-1 immunoreactivity in amacrine cell bodies in the INL. The scale bar for **G**-**L** is 45 μm. In **C** and **F** inner plexiform layer is abbreviated inner plexiform layer (IPL).

The expression of GAD_67_ and GAT-1 were evaluated in the *thy1*-CFP mouse retina. GAD_67_ and GAT-1 immunoreactivities were localized in numerous amacrine and displaced amacrine cells, with somata in the INL and GCL, respectively, and in densely distributed immunoreactive processes and puncta in all laminae of the IPL (Figure 7) [37,51-56]. Cell counts in the GCL showed that 12.4% and 9.2% of the CFP-containing cells in the GCL expressed GAD_67_ and GAT-1 immunoreactivity, respectively (Table 1). Conversely, 26.2% of the GAD_67_ immunoreactive cells and 17.1% of the GAT-1 immunoreactive cells in the GCL contained CFP (Table 1). GlyT-1 immunoreactivity, a neurochemical marker for glycinergic amacrine and displaced amacrine cells, did not colocalize with CFP-expressing cells in the GCL or INL (data not shown).

**Figure 8:**
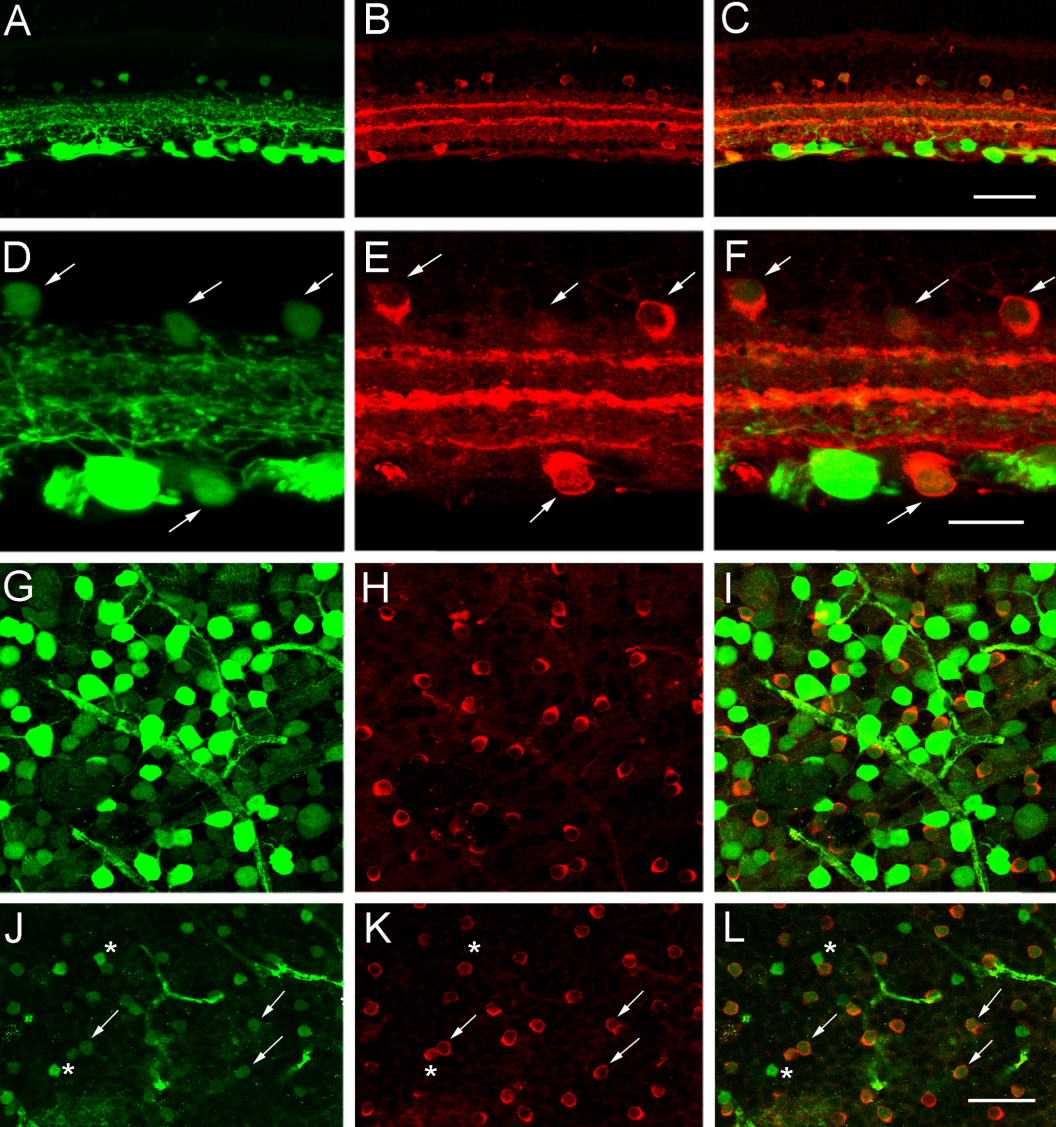
CFP expression colocalizes with ChAT immunoreactivity, a marker of cholinergic amacrine and displaced amacrine cells in the inner retina. **A:** A transverse section of peripheral retina shows cyan fluorescent protein (CFP) in numerous cell bodies in the ganglion cell layer (GCL), and weakly-CFP-expressing cell bodies in the proximal inner nuclear layer (INL). B: Choline acetyltransferase (ChAT) immunoreactivity is in amacrine and displaced amacrine cell somata in the INL and GCL, respectively. **C:** A merged image of **A** and **B** shows the colocalization of CFP and ChAT immunoreactivity in small, weakly CFP-expressing cell bodies in the INL and GCL. The scale bar for **A**-**C** is 45 μm. **D:** A higher magnification image of transverse retina shows CFP in ganglion cells in the GCL and in smaller, weakly CFP fluorescent cells in the INL and GCL (arrows). **E:** Same section as in **D** shows ChAT immunoreactivity in numerous amacrine and displaced amacrine cell somata in the INL and GCL (arrows). **F:** A merged image of **D** and **E** shows the colocalization of CFP expression and ChAT immunoreactivity in amacrine and displaced amacrine cells (arrows) in the INL and GCL. The scale bar for **D**-**F** is 25 μm. **G:** CFP is localized to brightly and weakly fluorescent cell bodies of various sizes in the GCL. Image is from a retinal wholemount located 1.5 mm from the optic nerve head in midperipheral nasal retina. **H:** ChAT immunoreactivity is in displaced amacrine cell somata in the GCL. **I:** A merged image of **G** and **H** shows colocalization of CFP expression and ChAT immunoreactivity in displaced amacrine cell bodies in the GCL. **J:** CFP is localized to weakly fluorescent cell bodies in the INL. Image is from a retinal wholemount located 1.5 mm from the optic nerve head in midperipheral nasal retina. **K:** The same region as in **J** shows ChAT immunoreactivity in amacrine cell somata in the INL. **L:** A merged image of **J** and **K** shows the colocalization of CFP and ChAT immunoreactivity in amacrine cell bodies in the INL (arrows). There are also a few small CFP somata that do not contain ChAT immunoreactivity in the INL (stars). The scale bar for **G**-**L** is 45 μm.

Finally, CFP-expressing amacrine and displaced amacrine cells were further classified using antibodies against the neurochemical marker ChAT, which immunolabel the cholinergic amacrine cell population [57]. These studies were based on the observed mirror-image symmetry of the small, weakly CFP-expressing cell bodies in the INL and GCL and the CFP-containing processes in laminae 2 and 4, which are indicative of the cholinergic amacrine cell population [57-63]. ChAT immunoreactivity was localized to amacrine and displaced amacrine cell bodies distributed in the INL and GCL, and their processes that narrowly stratified in laminae 2 and 4 of the IPL (Figure 8). Weak CFP expression was visualized in most ChAT immunoreactive somata in the INL and GCL, and in ChAT immunoreactive processes in the IPL (Figure 8C,F). Cell counts showed that 86.7% of the CFP-containing amacrine cells in the INL and 20.4% of CFP-containing cells in the GCL express ChAT immunoreactivity, respectively (Table 1). Conversely, all ChAT amacrine and displaced amacrine cells contained CFP. These data indicate that most CFP expression in amacrine cells is localized to the cholinergic amacrine cell population.

## Discussion

The retinal ganglion cell layer contains two major types of functionally distinct neurons: ganglion cells and displaced amacrine cells. These cells are often difficult to distinguish using morphological and histological techniques, especially in the mouse retina where many ganglion cells are small and measure about 10–12 μm in diameter [20,27]. An endogenous fluorescent marker that is expressed in all or the majority of ganglion cells would greatly simplify the task of distinguishing between the two cell types in studies that require the reliable identification of ganglion cells in vivo or in vitro. This study showed that CFP expression in the retina of the *thy1*-CFP transgenic mouse line (#23) developed by Feng et al. [5] is localized to the majority of ganglion cells, including their dendrites in the IPL and axons that form the optic nerve. Weak CFP expression was also expressed in the GCL and INL in cholinergic amacrine and displaced amacrine cells.

### Cyan fluorescent protein expression in the ganglion cell layer

CFP expression in the retina of the *thy1*-CFP transgenic mouse line is localized to numerous somata in the GCL, processes throughout the IPL, and axons in the nerve fiber layer, optic nerve head, and optic nerve. CFP-expressing cells in the GCL range in size from 6.12 to 19.74 μm in diameter and 1827±280 cells/mm^2^ to 3589±470 cells/mm^2^ in density, with an average cell density of 2914±312 cells/mm^2^, and 49,829±5,335 cells per retina (including the CFP-containing displaced amacrine cells–discussed in the section to follow). About 52% of the cells in the GCL express CFP, based on cell counts of CFP and DAPI labeling of the GCL in retinal whole mounts. Therefore, given that about 20% of CFP expression in the GCL is in displaced amacrine cells, about 80% of the CFP expressing cells in the GCL, or 40%–45% of the total neurons in the GCL of the *thy1*-CFP transgenic mouse line are ganglion cells. These data are in agreement with a study that extrapolated the number of ganglion cells in the GCL to be 40,000–44,000, by counting the number of axons in the optic nerve [24], and concluded that 41% of the neurons in the GCL are ganglion cells. Another study [64] reported an average of 54,600 ganglion cells per retina in the C57BL/6J retina, which is a higher value for the number of ganglion cells than what we have estimated. The overall variability in the total number of ganglion cells between the different C57BL/6J lines could be due to several factors including, but not limited to, genetic variability between inbred mouse strains, sampling methods, and tissue processing.

### Neurochemical characterization of cyan fluorescent protein expression in ganglion cells

Initial efforts to characterize CFP expression in the *thy1*-CFP mouse retina focused on colocalization studies with several neurochemical markers that have been localized to ganglion cells in prior studies. Experiments using antibodies to NF-L, a neuronal cytoskeletal component that is expressed in most retinal ganglion cells, but not amacrine cells [28-30], showed that 61.6% of the CFP cells expressed NF-L immunoreactivity. This was less than expected, given that in the hamster retina 88% of all retinal ganglion cells express NF-L immunoreactivity [29]. Perhaps this difference is due to the following factors: interspecies variations in neurofilament expression, which have been reported [28-30]; variations in immunostaining techniques with NF-L antibody [30]; or the localization of NF-L immunoreactivity primarily in axons and dendrites, but not in somata, which results in difficulties in visualizing labeled somata and undercounts of the number of NF-L immunolabeled ganglion cells.

Colocalization experiments with antibodies to the DNA-binding protein NeuN, which primarily label nuclei, resulted in better somatic labeling than that observed using NF-L antibodies. Most (95.4%) CFP cells in the GCL contained NeuN immunoreactivity. Since 71.3% of NeuN immunoreactive cells contained CFP, we expect that the remaining (approximately 30%) NeuN immunoreactive cells in the GCL that are CFP negative are displaced amacrine cells. This is in agreement with earlier studies indicating the expression of NeuN immunoreactivity by both ganglion and amacrine cells (Figure 6; [35]).

Similarly, CR antibodies labeled 90.4% of the CFP cells in the GCL, and conversely only 62.2% of the CR immunoreactive neurons in the GCL contained CFP. These findings suggest that many of the CR immunoreactive neurons in the GCL are displaced amacrine cells. CR expression by amacrine cells has been shown in several species including mouse [41-43].

Brn-3a is a POU domain regulatory transcription factor expressed in many retinal ganglion cells in mouse, rabbit, and monkey retinas [44,45]. In the GCL, there is significant overlap between the number of CFP-containing and Brn-3a immunoreactive cells; 95.3% of the Brn-3a immunoreactive cells express CFP. Conversely, 61.5% of all CFP-labeled cells in the GCL contain Brn-3a immunoreactivity. This is in general agreement with a study that reported Brn-3a immunoreactivity in approximately 35% of all GCL neurons [44].

### Neurochemical characterization of cyan fluorescent protein expression in amacrine cells

Small somata characterized by weak CFP expression were in the proximal INL and GCL. These CFP-containing somata in the INL were initially identified as amacrine cells based on their appearance, size, and distribution to the proximal INL. The small and round CFP-expressing somata in the GCL were also suggestive of their identity as displaced amacrine cells. Colocalization experiments with the general amacrine cell marker HPC-1 [47-49] confirmed the presence of CFP in amacrine and displaced amacrine cells in the INL and GCL, respectively. In the GCL, 8.8% of HPC-1 immunoreactive displaced amacrine cells contain CFP and 9.6% of the total CFP-expressing cells in the GCL are HPC-1 immunoreactive displaced amacrine cells. Most (90.4%) small HPC-1 immunoreactive somata did not contain CFP, and they account for about half of the somata in the GCL.

Amacrine cells can be further subdivided into two broad groups based on their primary fast neurotransmitter; about half of these cells were GABAergic and the rest were glycinergic. In our study, all CFP-containing amacrine cells in the INL were GABAergic based on their expression of the GABA synthetic enzyme GAD_67_ or the high affinity plasma membrane GABA transporter, GAT-1 [37,51-55,65]. In the INL, most of the CFP somata contain GAD_67_ (99.4%) and GAT-1 (98.9%) immunoreactivity, respectively. In the GCL, 12.4% of the CFP-expressing cells were GAD_67_ immunoreactive and 9.2% were GAT-1 immunoreactive. These cells were small and intermixed with those with medium and large somata, which are ganglion cells. There were also numerous small GAD_67_ (73.8% ) and GAT-1 (82.9%) immunoreactive somata that did not contain CFP. These cells are likely to be displaced amacrine cells based on their appearance, size, and neurochemistry. These observations, together with the experiments showing numerous small HPC-1 immunoreactive somata in the GCL that do not contain CFP, suggest that CFP is only expressed in a subgroup of displaced amacrine cells.

Cholinergic amacrine cells in both the INL and GCL expressed CFP, although at lower levels than ganglion cells. CFP expression by cholinergic amacrine and displaced amacrine cells was demonstrated using an antibody to ChAT, which robustly immunostains cholinergic amacrine cells [57]. All ChAT-immunoreactive amacrine cell somata in the INL and GCL contained CFP. Furthermore, cholinergic amacrine cells contain CR immunoreactivity [66], and the experiments using CR antibodies immunostained small and weak CFP-expressing amacrine and displaced amacrine cells, and processes in lamina 2 and 4 of the IPL, indicating weak expression of CFP in cholinergic amacrine cells.

Together, these findings show that CFP is expressed in a subset of GABAergic amacrine cells, the cholinergic amacrine cells, which contain GAD_67_ [51,67]. GABA-containing amacrine cells are characterized by wide-field morphology [67] and on this basis the CFP-containing amacrine cells in this mouse line are wide-field amacrine cells.

### Ectopic transgene expression in transgenic mouse lines

Transgenic mice with reporter genes in retinal neurons are important tools for defining retinal morphology and circuitry. The expression of the reporter gene can be targeted using cell-type specific promoter sequences, such as the promoter sequence of the *thy1* gene that was used in *thy1*-CFP mice to target CFP expression to ganglion cells in the retina. However, often expression differs from, or extends beyond, the expected cellular distribution of the gene, as in the case of CFP expression in the cholinergic amacrine cell population in the *thy1*-CFP mouse line. Similarly, other *thy1* transgenic mouse lines developed by Feng et al. [5] express GFP and its variants in several other cell types, including amacrine, displaced amacrine, and bipolar cells. Alternatively, expression of the transgene may be entirely ectopic, as in a recently developed CD44 transgenic line with multiple labeled amacrine and ganglion cells, but not Müller cells as expected [68]. Another example is the ectopic GFP expression in the somatostatin receptor 2 (sstr2)-EGFP BAC mouse line (unpublished observations). In this line, GFP is expressed in several amacrine cell types, but not in photoreceptor, horizontal, or bipolar cells, as expected from immunohistochemical and electrophysiological findings [69,70]. However, since transgene expression is usually stable and reproducible in these animal lines, careful characterization of transgene expression in retinal neuronal populations will undoubtedly yield useful tools for future studies.

In conclusion, the present study reports the extensive expression of CFP in most ganglion cells and their processes in the *thy1*-CFP transgenic mouse line [5]. Cholinergic amacrine and displaced amacrine cells also express CFP, but do so at lower levels. The *thy1*-CFP transgenic mouse line will be of value for studies requiring the rapid and reliable identification of the ganglion cell population, including studies of ganglion cell retinopathy.
